# Hormone Anchored Metal Complexes. 1. Synthesis, Structure, Spectroscopy and *In Vitro* Antitumor Activity of Testosterone Acetate Thiosemicarbazone and its Metal Complexes

**DOI:** 10.1155/MBD.1999.177

**Published:** 1999

**Authors:** Anupa Murugkar, Bindu Unnikrishnan, Subhash Padhye, Ramesh Bhonde, Simon Teat, Evangelia Triantafillou, Ekkehard Sinn

**Affiliations:** 1 Department of Chemistry University of Pune Pune 411007 India; 2 National Centre for Cell Science Ganeshkhind Pune 411007 India; 3 CLRC Daresbury Cheshire WA4 4AD UK; 4 Department of Chemistry University of Hull Hull HU6 7RX UK

## Abstract

Testosterone acetate thiosemicarbazone (TATSC, 17-β-hydroxyandrost-4-one acetate thiosemicarbazone) was synthesized and characterized by single crystal X-ray structure determination. The copper and platinum complexes of this steroid derivative were synthesized and characterized by spectroscopy and electrochemiatry. The in vitro activity of these compounds against human breast cancer cell line MCF-7 was tested. The highest activity was found for the [Pt(TATSC)Cl_1_] followed by [Cu(TATSC)Cl_2_] and the ligand in compariosn with cisplatin.


### HORMONE ANCHORED METAL COMPLEXES. 1. SYNTHESIS, STRUCTURE, SPECTROSCOPY AND *IN VITRO* ANTITUMOR ACTIVITY OF TESTOSTERONE ACETATE THIOSEMICARBAZONE AND ITS METAL COMPLEXES

Anupa Murugkar[1], Bindu Unnikrishnan[1], Subhash Padhye*[1], Ramesh Bhonde[2], Simon Teat[3], Evangelia Triantafillou[4] and Ekkehard Sinn[4]

[1] Department of Chemistry, University of Pune, Pune-411007, India
[2] National Centre for Cell Science, Ganeshkhind, Pune-411007, India
[3] CLRC Daresbury, Cheshire WA4 4AD, UK
[4] Department of Chemistry, University of Hull, Hull HU6 7RX, UK

**Abstract**

Testosterone acetate thiosemicarbazone (TATSC, 17-β-hydroxyandrost-4-one acetate thiosemicarbazone) was synthesized and characterized by single crystal X-ray structure determination. The copper and platinum complexes of this steroid derivative were synthesized and characterized by spectroscopy and electrochemiatry. The in vitro activity of these compounds against human breast cancer cell line MCF-7 was tested. The highest activity was found for the [Pt(TATSC)$Cl_2$] followed by [Cu(TATSC)$Cl_2$] and the ligand in compariosn with cisplatin.

**Introduction**

Transporting a cytotoxic drug selectively to the malignant cells without damaging the cells of the host organism is the desirable goal in the drug design for anticancer therapy. This task is made difficult because the morphological and biochemical differences between the malignant and normal cells are often minute. However, the presence of steroid hormone receptors found in high concentration in malignant cells of breast, ovary and prostate than in most of the normal cells offer excellent opportunities for such selective targeting by attaching a cytotoxic drug to the receptor binding carrier molecules.[1] The cytotoxic agents that have so far been linked in such a manner include nitrosoureas,[2-4] nitrogen mustards,[2,5] epoxides, aziridines, DNA intercalators and platinum complexes.[6-13]

In the present paper we report the synthesis and molecular structure of new steroidal cisplatin conjufates and a preliminary evaluation of their antiproliferative activity against human breast cancer cell line, viz. MCF-7.

**Experimental**

All the chemicals used in the synthesis of ligand and its metal complexes were of AR grade. Solvents used in the synthesis and spectroscopic studies were distilled prior to their use.

Testosterone acetate (Sigma Chemicals), $K_2PtCl_4$ (John Baker, USA), $CuCl_2.2H_2O$ (Qualigens), thiosemicarbazide (CDH) were used as supplied. Cisplatin was a gift from Dr. Kasabekar. Thiosemicarbazide hydrochloride was prepared by adding 1 ml of concentrated hydrochloric acid to a slurry of 1.1 g of powdered thiosemicarbazide in ethanol. The white precipitate was isolated by filtration with adequate washings with cold ethanol and water to remove excess acid. It was recrystallized from water and used after drying in vacuum at room temperature.

*Testosteroneacetate thiosemicarbazone (TATSC)* :

For the synthesis of the thiosemicarbazone ligand, 1.0 g of testosterone acetate (TA) was dissolved in acetone to which was added 0.39 g TSC.HCl in a 1:1 molar ratio dissolved in minimum amount of distilled water. The resulting reaction mixture was heated at 60°C with constant stirring for 6-8 hr during which the colour of the mixture changed from colourless to intense yellow. After completion of the reaction the solvent was stripped off on the rotavapor till yellow crystalline TATSC separated out. It was washed with cold water and dried in vacuum. All attempts to grow crystals suitable for single crystal X-ray structure determination via a normal diffractometer were unsuccessful. A crystal suitable for X-ray diffraction studies via high intensity synchrotron radiation was grown as pale yellow plate grown from acetonitrile solvent by slow evaporation.

*Metal Complexes*

These were prepared by reacting the ligand TATSC and corresponding metal chlorides in a 1:1 molar ratio in acetonitrile solvent at 40°C with constant stirring for 8 hr. $K_2PtCl_4$ was used as the satrting material in the synthesis of the platinum complex whereas the copper complex was synthesized using $CuCl_2.2H_2O$.

*Instruments* :

Elemental analyses were carried out in the Microanalytical Laboratories of the Universities of Pune and Hull. The magnetic susceptibilities of the metal complexes were measured at 300K on a Faraday Balance. IR

spectra were recorded as KBr discs in the range 4500 - 450 $cm^{-1}$ on a Perkin-Elmer-1615 FTIR spectrophotometer. Electronic spectra were recorded on a Genesys-2 UV-VIS-NIR spectrophotometer in the range 200-1100 nm. Cyclic voltammetric (CV) measurements were made in acetonitrile solvent on a BioAnalytical System BAS CV-27 with XY-recorder using Pt as working electrode against SCE and a Pt wire as an auxillary electrode with tetraethylammonium perchlorate (TEAP) as a supporting electrolyte.

*X-ray crystal structure determination* :

The measurements were made on a Bruker AXS SMART CCD area detector diffractometer at Station 9.8 of the CLRC Daresbury Laboratory synchrotron radiation source.[14] The data was collected[15] at 150K using ω rotation scans with narrow frames and a wavelength of 0.6849 on a yellow crystal of TATSC, $C_{22}H_{32}N_3O_2S$ dimension 0.16 x 0.10 x 0.02 mm. A least squares refinement of the data gave an orthorhombic cell of TATSC. Full-matrix least-squares refinement of the setting angles yielded a orthorhombic cell with a = 8.0940(6) Å, b = 8.1463(6) Å, c = 33.100(3) Å, V = 2182.5(3) $Å^{3}$. For Z = 4 and F.W. = 402.57, the calculated density is 1.225 $g/cm^3$. Based on systematic absences, packing considerations, statistical analysis of intensity distribution and the solution and refinement of structures, the space group was determined to be $P2_12_12_1$. Of the 15118 reflections collected, 4276 are unique ($R_{int}$ = 0.898). **Table 3** shows a summary of crystal data and X-ray analysis information for TATSC and the selected bond lengths and bond angles are listed in **Table 4**.

The structure was solved by direct methods.[16] Hydrogen atoms showed clearly on difference Fourier maps. These were geometrically placed and fixed. The non-hydrogen atoms were refined anisotropically. Neutral atom scattering factors were taken from Cromer and Waber.[17] Full-matrix lwast-squares refinements[16,18] gave weighted and unweighted agreement factors of $R_1$ = 0.0742, $wR_2$ = 0.1550 (for all data above $I>2\sigma$) and $R_1$ = 0.1110, $wR_2$ = 0.1716 (for all data) respectively.

*Anticancer Activity Assay*

The MCF-7 human breast adinocarcinoma cell line was obtained from the National Centre for Cell Science (NCCS), Pune. The cells were cultured as monolayers and maintained in a growth medium (Eagles' Minimum Essential Medium (MEM(E))) consisting of MEM(E) supplemented with 10% FCS, 50μg/ml streptomycin and 50 μg/ml glutamin. Stock cultures were harvested from 25 $cm^2$ disposable tissue culture treated flasks and maintained in the incubator with a constant humidified atmosphere of 5% $CO_2$ and 95% air at 37°C.

The stock solutions of TATSC and its copper and platinum complexes were prepared as 1mg/ml solutions in DMSO, filtered through 0.22 μm filters and stored in bottles at 8°C. The 0.5 mg/ml stock solution of cisplatin was made in normal saline, filtered and stored at 8°C. A neutral red solution was prepared by dissolving 1 mg neutral red (NR) in 20 ml of MEM(E), warmed and centrifuged at 90 g for 10 min for removing any neutral red crystals before filtration. De-staining solution was prepared with 1% acetic acid, 50% ethanol and 49% distilled water v/v according to an established protocol.[19] A Spectrochem-250 ELISA reader was used to read the final absorbances.

Before performing the neutral red assay, a preliminary range finding analysis of the potential toxicity of the test compounds was conducted wherein the highest tolerated dose (HTD) was defined as the highest concentration of the compound that is tolerated by the cells with minimal alterations in the morphology. For this analysis, the cells were seeded in 24-well plate at a density of 2 x $10^5$ cells/ml. After 24 hr incubation, 100 μl of the medium was removed from each well except control and to each sample (in triplicate), 100 μl of the test compound (at different concentrations) was added. Quantitation of the viable cells was carried out by direct counting with a hemocytometer using trypan blue after 24 and 48 hours.

Since the active compounds were cytotoxic even at the concentration of 0.1 mg/ml, a much lower concentration range was explored and the cell viability was determined by a neutral red assay using a 96 well plate method.[19]

**Results and Discussion**

The analytical data including elemental analyses, electronic transitions and redox potentials of the complexes is given in **Table 1**. All the complexes are found to be diamagnetic at room temperature.

The assignment of the significant vibrational bands of the ligand and its metal complexes are reported in **Table 2**. The IR spectrum of testosterone acetate thiosemicarbazone (TATSC) exhibits absorption bands at 3425 and 3245 $cm^{-1}$ due to symmetric and asymmetric ν(NH) stretching vibrations which are shifted to the higher frequency side on metal complexation. The C(3) C=O frequency in TA was replaced by C=N on condensation with thiosemicarbazone pharmacophore. Other absorptions which are affected upon complex formation include those due to ν(N-N) stretch at 1053 $cm^{-1}$ and the ν(C=N) stretch at 1587 $cm^{-1}$ indicating the involvement of the azomethine nitrogen in metal coordination. The assignment of the bands involving the C=S group is uncertain and difficult due to its mixing with other frequencies over a wide range (1300-700 $cm^{-1}$).[20,21] Unaltered frequencies on complexation are assigned to the methyl group (carbon portion) of the steroidal nucleus (2920 $cm^{-1}$) and the acetate group at C-17 position (1730 $cm^{-1}$) respectively.

**Table 1.** Analytical data on [Cu(TATSC)$Cl_2$] and [Pt(TATSC)$Cl_2$] including elemental analyses, electronic transitions and redox potentials.

| Compound | Elemental Analysis | | | Electronic Spectra[a] | $E_{1/2}$[a] (V) | |
|---|---|---|---|---|---|---|
| | %C | %H | %M | $cm^{-1}$ | $M^{2+}/M^{1+}$ | L |
| TATSC | 64.76 | 8.38 | -- | 27932, 25000, 24096 | -- | -1.05 |
| | (65.50) | (8.25) | -- | | | |
| [Cu(TATSC)$Cl_2$] | 42.54 | 5.88 | 10.66 | 22471, 23474, 25000, | +0.18 | -0.77 |
| | (42.98) | (5.75) | (11.07) | 19153, 15432 | | |
| [Pt(TATSC)$Cl_2$] | 39.11 | 4.78 | 28.90 | 28571, 25974, 22222, | -- | -0.98 |
| | (39.42) | (4.82) | (29.58) | 20202, 19047 | | |

[a]In DMSO; Parentheses show calculated values; L = ligand based peaks

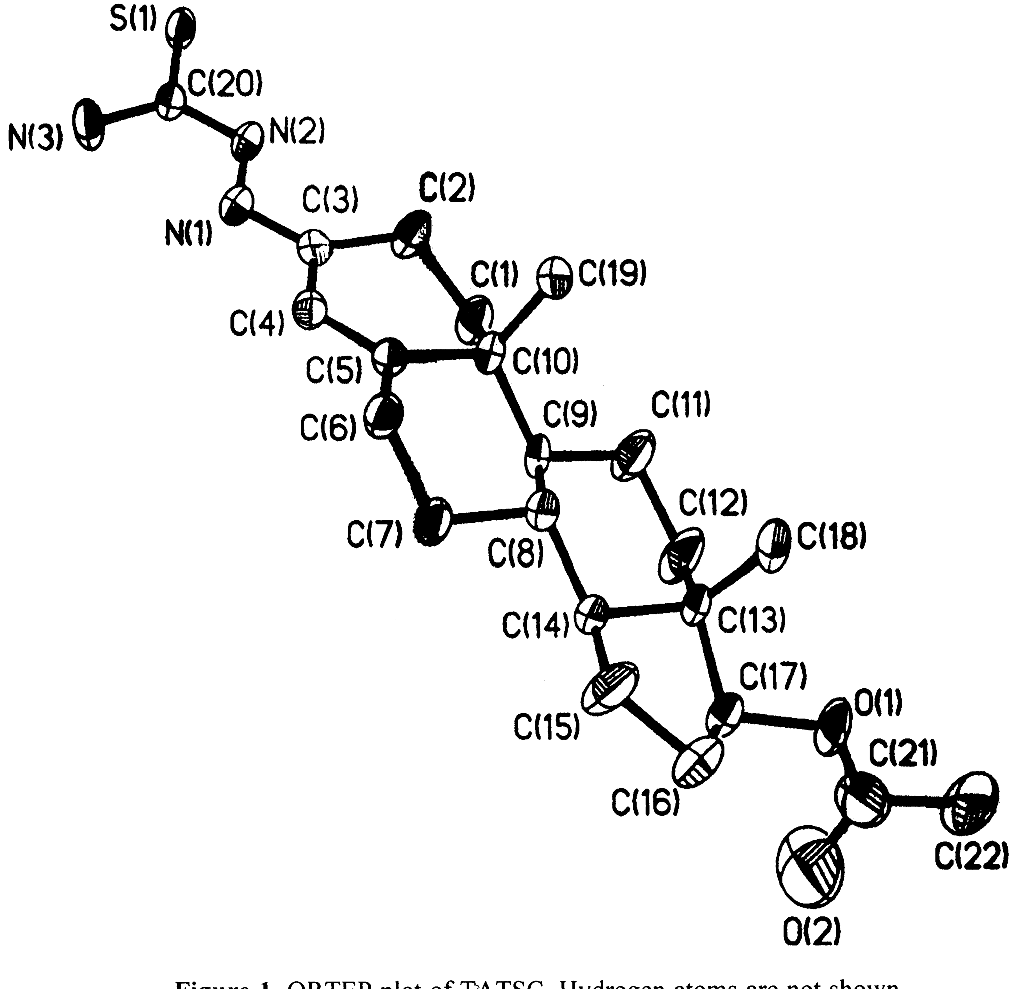

**Figure 1.** ORTEP plot of TATSC. Hydrogen atoms are not shown.

The copper complex shows bands at 15432 and 19153 $cm^{-1}$ due to d-d transitions assigned to $d_{xy} \rightarrow d_{z^2}$ and $d_{xy} \rightarrow d_{xz}$ respectively. The bands corresponding to LMCT are observed at 22471, 23474 and 25000 $cm^{-1}$. The platinum complex shows bands at 20202 and 19047 $cm^{-1}$ assigned to $^1A_{1g} \rightarrow {}^3A_{2g}$. A broad band at 22222 $cm^{-1}$ in this compound may be due to mixing of d-d and CT bands. Two ligand-based peaks are also observed for this compound at 28571 and 25974 $cm^{-1}$ respectively.

**Table 2**. Significant IR bands of TATSC and its metal complexes*

| Compound | ν($CH_3COO$) | ν(C=O) | ν(-NH) Sym. | Asym. | ν(C=N) | ν(C=S) | ν(N-N) |
|---|---|---|---|---|---|---|---|
| TA | 1700 | 1669 | -- | -- | – | – | – |
| TATSC | 1729 | – | 3425 | 3245 | 1587 | 956 | 1053 |
| [Cu(TATSC)$Cl_2$] | 1732 | – | 3342 | 3200 | 1540 | – | 1047 |
| [Pt(TATSC)$Cl_2$] | 1714 | – | 3336 | 3228 | 1556 | – | 1045 |

*All values in $cm^{-1}$, TA = Testosterone acetate

**Table 1** gives the cyclic voltammetric data on the ligand and its complexes in acetonitrile using 0.1 M TEAP vs SCE at 100 mV/s. The CV profile of the ligand in acetonitrile exhibits a broad, irreversible peak at -1.05 V with no anodic counterpart which can be attributed to the reduction of C=S (thione)[22] chromophore. On complexation with copper a reversible peak centered at +0.18 V can be seen which is due to $Cu^{2+}/Cu^{1+}$ redox couple. An additional irreversible peak observed at -0.77 V is assignable to the ligand. The shifted ligand potential on complexation indicates easier reduction of the C=S moiety. In the platinum complex no metal-based peak is observed but an irreversible peak at -0.98 due to the ligand reduction can be seen.

A perspective view of TATSC is shown in **Figure 1**. Crystallographic details of the two compounds are given in **Table 3** while selected bond distances and bond angles are listed in **Table 4**.

**Table 3**. Data collection parameters for TATSC

| | |
|---|---|
| Formula | TATSC ($C_{22}H_{32}N_3O_2S$) |
| M | 402.57 |
| Crystal system | Orthorhombic |
| Space group | P212121 |
| a/Å | 8.0940(6) |
| b/Å | 8.1463(6) |
| c/Å | 33.100(3) |
| α/° | 90 |
| β/° | 90 |
| γ/° | 90 |
| Z | 4 |
| V/$Å^{-3}$ | 2182.5(3) |
| $\mu_{calc}/cm^{-1}$ | 0.017 |
| $D_c$/ $gcm^3$ | 1.225 |
| F(000) | 868 |
| Crystal dimensions/mm | 0.16 x 0.10 x 0.02 |
| Radiation (λ/Å) | 0.6849 |
| θ range for data collection/° | 2.48 to 25 |
| Scan mode | ω rotations with narrow range |
| hkl ranges | -9 to 9; -9 to 10; -36 to 40 |
| Reflections collected | 15118 |
| Independent reflections | 4276 ($R_{int}$ = 0.0898) |
| Refinement method | Full-matrix least-squares on $F^2$ |
| R(F) | 0.1110 |
| wR(F) | 0.1716 |
| Largest difference peak and hole/$Å^{-3}$ | 0.544 and -0.250 |

**Table 4**. Interatomic distances (Å) and bond angles (°) in TATSC

| Distances / Angstrom | | | |
|---|---|---|---|
| C(3) - N(1) | 1.302(5) | N(1) - N(2) | 1.376(5) |
| N(2) - C(20) | 1.296(5) | C(20) - S(1) | 1.672(4) |
| C(20) - N(3) | 1.356(5) | | |
| **Angles/Degree** | | | |
| C(2) - C(3) - N(1) | 125.8(4) | N(2) - C(20) - S(1) | 119.1(3) |
| C(3) - N(1) - N(2) | 115.5(4) | N(1) - N(2) - C(20) | 117.0(3) |

It can be seen from the structure of TATSC that the molecule crystallizes as the E isomer with the N(2) litrogen and S(1) sulfur on one side of the C(20)-N(2) bond. The packing diagram shows no intermolecular hydrogen bonding contacts.

A comparison of the bond distances with other E isomers including 4N-substituted formyl- and acetylpyridine thiosemicarbazones and α-ketobutyric acid thiosemicarbazone shows that in the present compound the C(3)-N(1) bond is slightly longer (0.02 Å) while C(20)-S(1) distance is shorter by 0.23 Å which probably results in a stronger C(20)-N(2) bond. The N(1)-N(2) bond is the longest observed than in any TSC ligand. The bond angle C(20)-N(2)-N(1) is smaller (117.0°) than other E isomers although it is smaller when compared with the Z isomers.[23] The bond angles C(3)-N(1)-N(2) (115.5°) and N(2)-C(20)-S(1) (119.1(3)°) are comparable with those observed in other E isomers. The observation that metal complexation results in the transformation of the E isomer into the Z configuration with enhanced biological activity suggest that such E→Z conversion might be crucial for the biological effects of the thiosemicarbazone ligands.

All the synthesized compounds were tested on human breast cancer cell line, MCF-7 which showed comparable cytotoxicity at identical concentration (**Figure 2**) as the standard cisplatin compound.

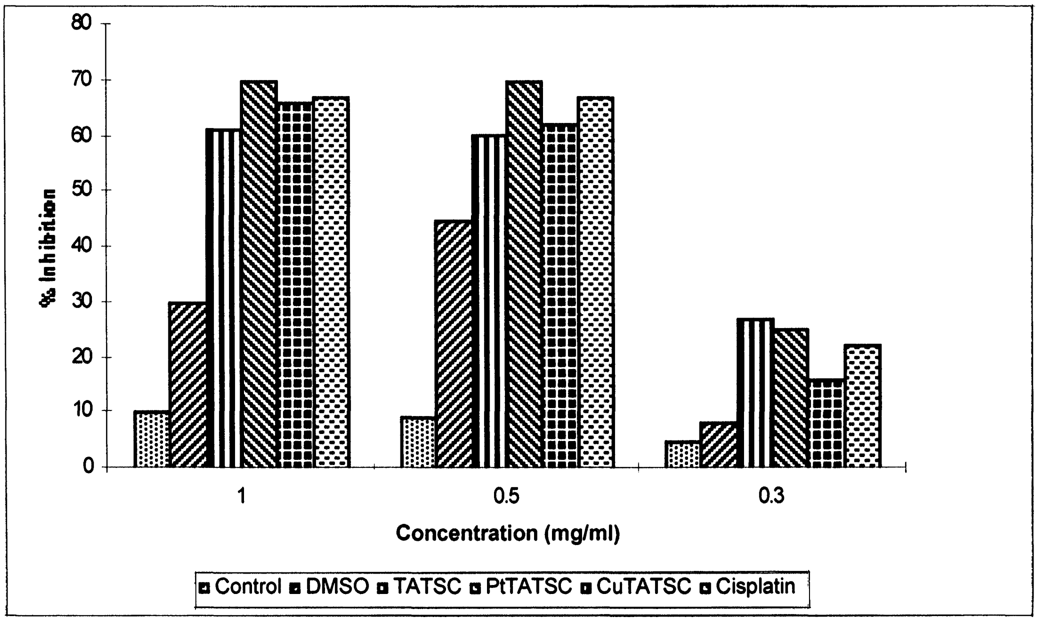

**Figure 2**. Concentration dependent growth inhibition of MCF-7 (Human Breast Cancer Cell Line)

For the TATSC ligand an increase in the cytotoxicity was observed on metal complexation which can be explained on the basis of conversion to the preferred Z configuration. Thiosemicarbazone pharmacophores are known to inhibit DNA synthesis via inhibition of the enzyme RDR and to inflict DNA damages. These aspects are under investigation presently.

**Acknowledgements**
AM would like to thank CSIR, New Delhi, India for the financial assistance in the form of Senior Research Fellowship (SRF) while SP would like to acknowledge support from British Council in the form of a HE Link visit support.

**References**
1. E.von Angerer, " The Estrogen Receptor as a target for Rational Drug Design", (1997).
2. G. Leclercq, N. Devleeschouwer, N. Legros, In H.T.Mourisden, T. Palshof eds., "Breast Cancer - Experimental and Clinical Aspects", Oxford, Pergamon, 287 (1980).
3. B. Hartley-Asp, *Prostate*, **5**, 93 (1984).
4. T. Kubota, K. Ishibiki, O. Abe, H. Kosano, N. Oshawa and R.M.Hoffman, *Anticancer Res.*, **13**, 935 (1993).
5. H. Hamacher, *Arch. Pharm.* (Weinheim) **311**, 184 (1978).
6. V.K.Kansal, P. Potier, *Tetrahedron*, **42**, 2389 (1986).
7. N. Knebel, E.von Angerer, *J.Med.Chem.*, **31**, 1675 (1988).
8. E.von Angerer, H. Birnbock, N. Knebel, *Anticancer Drug Res.*, **4**, 21 (!989).
9. N.G. Knebel, E.von Angerer, *J.Med.Chem.*, **34**, 2124 (1991).
10. S.von Angerer, E. Seidl, A. Mannschreck, E.von Angerer, W. Wiegrebe, *Anticancer Drug Design*, **9**, 25 (1994).
11. R. Ambros, S.von Angerer, W. Wiegrebe, *Arch. Pharm.* (Weinheim) **321**, 743 (!988).
12. R. Ambros, M.R. Schneider, S.von Angerer, *J.Med.Chem.*, **33**, 153 (1989).
13. T.Polossek, R. Ambros, S.von Angerer, G. Brandl, A. Mannschreck, E.von Angerer, *J.Med.Chem.*, **35**, 3537 (1992).
14. A New High Flux Chemical and Materials Crystallography Station at the SRS Daresbury : Part 1. Design, Commissioning and Test Results - R.J.Cernik, W.Clegg, C.R.A. Catlow, G. Bushnell-Wye, J.V. Flaherty, G.N. Greaves, M. Hamichi, I.D.Borrows, D.J.Taylor and S.J.Teat, *J. Synchrotron Rad.*, **4**, 279 (1997).
15. W.Clegg, M.R.J. Elsegood, S.J.Teat, C. Redshaw and V.C. Gibson, *J.Chem.Soc.Dalton Trans.*, 3037 (1998).
16. C.J.Gilmore, MITHRIL - an integrated direct methods computer program (incorporates MULTAN), University of Glasgow, *J.Appl.Cryst.*, **17**, 42 (1984).
17. TEXSAN-TEXRAY Structure Analysis Package, Molecular Structure Corporation (1985).
18. D.T.Cromer, J.T.Waber "International Tables for X-ray Crystallography,", Vol. IV, The Kynoch Press, Birmingham, England, Table 2.2 A (!974).
19. H. Babich and E. Borenfreund, *ATLA*, **18**, 129 (1990).
20. S.Padhye, G.B.Kauffman, *Coord.Chem.Rev.*, **63**, 127 (1985).
21. S.Padhye, R. Chikate, A. Kumbhar, S.J.Shallon and M.P.Chitnis, *Biol.Metals*, **5**, 67 (1992).
22. (a) P.Sonawane, R.Chikate, A.Kumbhar, S.Padhye and R. Doedens, *Polyhedron*, **13**, 395 (1994).
(b) P.Sonawane, A.Kumbhar, S.Padhye and R.J.Butcher, *Transition Met.Chem.*, **19**, 277 (1994).
23. D.X.west, G.A.Bain, R.J.Butcher, J.P.Jasinski, Yu Li, R.Y.Pozdniakiv, J. Valdes-Martinez, R.A. Toscano and S.H.Ortega, *Polyhedron*, **115**, 665 (1996).

**Received: March 31, 1999 - Accepted: April, 30, 1999 - Received in revised camera-ready format: May 21, 1999**

